# Persistence of balsam fir and black spruce populations in the mixedwood and coniferous bioclimatic domain of eastern North America

**DOI:** 10.1002/ece3.5069

**Published:** 2019-04-13

**Authors:** Yassine Messaoud, Venceslas Goudiaby, Yves Bergeron

**Affiliations:** ^1^ NSERC/UQAT/UQAM Industrial Chair in Sustainable Forest Management, Institut de recherche sur les forêts (IRF) Université du Québec en Abitibi‐Témiscamingue Rouyn‐Noranda Quebec Canada

**Keywords:** balsam fir, black spruce, climate change, coniferous domain, mixedwood domain, natural regeneration

## Abstract

The boreal ecocline (ca 49°N) between the southern mixedwood (dominated by balsam fir) and the northern coniferous bioclimatic domain (dominated by black spruce) may be explained by a northward decrease of balsam fir regeneration, explaining the gradual shift to black spruce dominance. 7,010 sample plots, with absence of major disturbances, were provided by the Quebec Ministry of Forest, Fauna, and Parks. The regeneration (sapling abundance) of balsam fir and black spruce were compared within and between the two bioclimatic domains, accounting for parental trees, main soil type (clay and till) and climate conditions, reflected by summer growing degree‐days above 5°C (GDD_5), total summer precipitation (May–August; PP_MA). Parental trees and soil type determined balsam fir and black spruce regeneration. Balsam fir and black spruce, respectively, showed higher regeneration in the mixedwood and the coniferous bioclimatic domains. Overall, higher regeneration was obtained on till for balsam fir, and on clay soils for black spruce. GDD_5 and PP_MA were beneficial for balsam fir regeneration on clay and till soils, respectively, while they were detrimental for black spruce regeneration. At a population level, balsam fir required at least 28% of parental tree basal area in the mixedwood, and 38% in the coniferous bioclimatic domains to maintain a regeneration at least equal to the mean regeneration of the whole study area. However, black spruce required 82% and 79% of parental trees basal area in the mixedwood and the coniferous domains, respectively. The northern limit of the mixedwood bioclimatic domain was attributed to a gradual decrease toward the north of balsam fir regeneration most likely due to cooler temperatures, shorter growing seasons, and decrease of the parental trees further north of this northern limit. However, balsam fir still persists above this northern limit, owing to a patchy occurrence of small parental trees populations, and good establishment substrates.

## 1 INTRODUCTION

Tree species exhibit greater sensitivity to climate conditions when they are located at their northern range limit (Fang & Lechowicz, 2006; Kharuk et al., 2013; Kremenetski, Sulerzhitsky, & Hantemirov, 1998; Merlin, Duputie, & Chuine, 2018). Conversely, the location of the northern limit of a species' distribution is sensitive to minor changes in climate, which can result in a reduction or an increase in regeneration potential (James, 2011; Kullman, 1993; Wright, Nguyen, & Anderson, 2016; Zhao et al., 2012). Frost and drought can also kill or cause heavy damages to seedlings (Gurney, Schaberg, Hawley, & Shane, 2011; Langvall, Nilsson, & Örlander, 2001; Langvall & Örlander, 2001; Nlungu‐Kweta, Leduc, & Bergeron, 2014), thereby reducing establishment and survival.

Nevertheless, other factors that may hamper regeneration include seed supply and availability of suitable germination sites (Caspersen & Saprunoff, 2005; Garcia, Banuelos, & Houle, 2002; Newsome, Brown, & Nemec, 2016). At their northern distribution limits, species produce less viable seeds due to reduced occurrence and duration of suitable climate conditions (Grigorieva & Moiseev, 2018; Pigott, 1992; Sirois, 2000; Tremblay, Bergeron, Lalonde, & Mauffette, 2002). Furthermore, ecological conditions that are favorable for seed germination might not necessarily be good for seedling survival (Camill et al., 2010; Dovciak, Reich, & Frelich, 2003; LePage, Canham, Coates, & Bartemucci, 2000; Schupp & Fuentes, 1995). Indeed, soil fertility is lower in colder climates because low soil temperatures inhibit organic matter decomposition and decrease soil evaporation, thereby decreasing nutrient availability (Shugart, Leemans, & Bonan, 1992; Trugman et al., 2016). Suitable germination and establishment sites typically become increasingly less frequent when moving toward the northern limit of a species' distribution, thus limiting or delaying regeneration despite increased seed production due to climate warming (Camill et al., 2010; Kroiss & HilleRisLambers, 2015; Lloyd, 2005; Suarez, Binkley, Kaye, & Stottlemyer, 1999).

In addition, most studies focusing on links between species distributions and regeneration dynamics have been conducted in areas where species have reached their distribution limits (Dyderski, Paź, Frelich, & Jagodziński, 2018; Engelmark, Bergeron, & Flannigan, 2000; Hogg, 1994; Zeng et al., 2016). In contrast, only a few studies have concentrated on the dynamics of the boundary between two types of forest ecosystems (Diochon, Rigg, Goldblum, & Polans, 2003; Fisichelli, Frelich, & Reich, 2013,2014; Goldblum & Rigg, 2002). This boundary represents major transitions from the temperate to boreal biome at mid‐ and higher latitudes and elevations, together with regions where a given species does not necessary reach its distributional limits, but nevertheless decreases in abundance toward higher latitudes or altitudes.

The global boreal zone is geographically situated north of 50° latitude (Shugart et al., 1992), but extends below this latitude in Eastern Canada, where it is considered as the southernmost in the world, apart from the altitude (Pouliot, 1998). This location encompasses western Quebec and eastern Ontario, *that is,* the largest provinces of central Canada. In Quebec, the area is composed of two bioclimatic domains that are characterized by different late‐successional species in mesic sites: the southern balsam fir (*Abies balsamea* (L.) Mill.)—paper birch (*Betula papyrifera* Marsh.) bioclimatic domain (hereafter, referred to as mixedwood forest) and the northern black spruce (*Picea mariana* (Mill.) BSP—feather moss bioclimatic domain (hereafter, referred to as coniferous forest; Saucier, Grondin, Robitaille, & Bergeron, 1998). Trembling aspen (*Populus tremuloides* Michx.), paper birch and jack pine (*Pinus banksiana* Lamb.) are abundant immediately after fire in both bioclimatic domains.

Balsam fir and black spruce have contrasting ecological traits. Balsam fir is more shade‐tolerant but less cold‐tolerant than black spruce. Balsam fir has no seed bank, while black spruce cones containing seed remain in the canopy for several years (Messaoud, Bergeron, & Asselin, 2007). Further, balsam fir is more fire intolerant compared to black spruce given that the former has a thinner bark (Bakuzis & Hansen, 1965). Thus, large and severe fires are detrimental to balsam fir regeneration, with seed availability strongly dependent up on living parental trees in areas free of fire. In addition, black spruce is more adapted to soil water–logging conditions compared to balsam fir (Messaoud, Bergeron, & Leduc, 2007).

The objective of the study was to determine whether reduced regeneration potential of balsam fir in the coniferous compared to the mixedwood bioclimatic domain plays a role in the location of the boundary between the two forest types.

Indeed, lower regeneration was expected for balsam fir in the coniferous domain, where climate conditions that are favorable to seedling establishment and survival occur less frequently than in the mixedwood domain. Given that some balsam fir populations still persist in the coniferous domain, we also verify to what extent the maintenance of such populations is linked with parental trees and regeneration potential driven by climate and site conditions.

## 2 MATERIALS AND METHODS

### 2.1 Study area and sampling design

**Figure 1:**
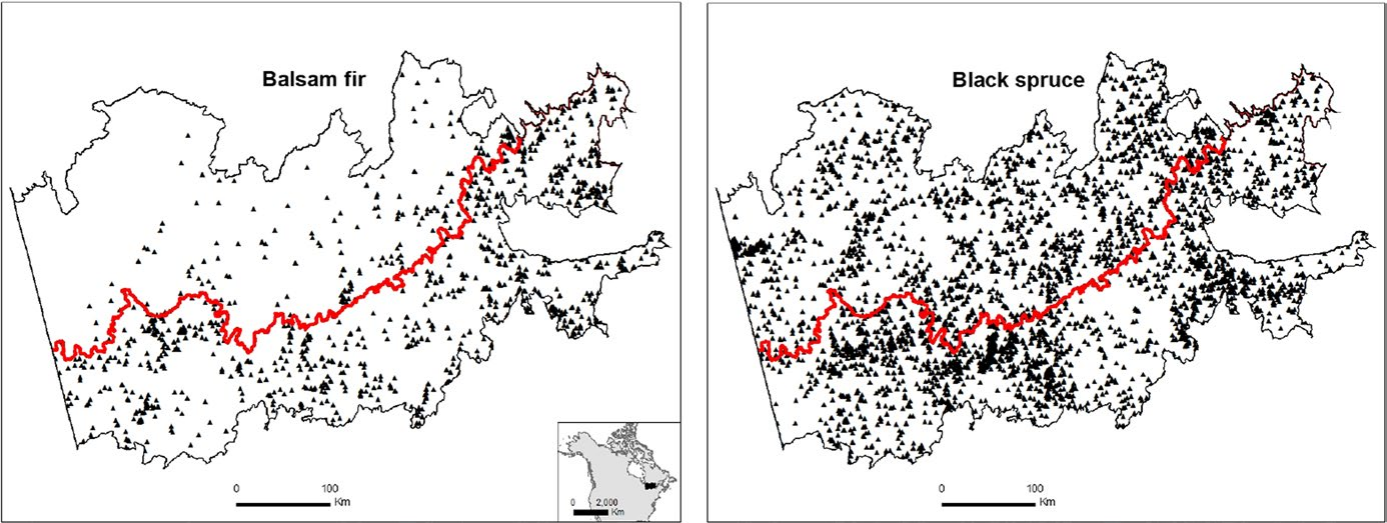
Study area showing the sample plots for balsam fir and black spruce stands that were established for the collection of forest inventory data by *Ministère de la Forêt, de la Faune et des Parcs du Québec* (MFFPQ). The bold red line that runs from east to west depicts the boundary between the southern mixedwood and northern coniferous bioclimatic domains in western Quebec (after Saucier et al., 1998)

The study area was located in northwestern Quebec (Canada) and is a part of the Quebec and Ontario Clay Belt, which was formed by lacustrine deposits left by proglacial lake Barlow‐Ojibway (Veillette, 1994; Figure 1). Elevation generally varies between 300 and 400 m asl, and low hills are scattered in an otherwise flat landscape.

The climate is continental, with cold winters and warm summers. Mean annual temperature in the mixedwood bioclimatic domain is 1.2°C (Amos meteorological station; 48°34′N, 78°07′W; 310 m elevation). Mean temperature of the coldest (January) and warmest (July) months are −17.3°C and 17.2°C, respectively. Total annual precipitation is 918 mm, of which 248 mm falls as snow. In the coniferous bioclimatic domain, mean annual temperature is −0.7°C (Matagami meteorological station; 49°46′N, 77°49′W; 281 m elevation). Mean temperature of the coldest (January) and warmest (July) months are −20.0°C and 16.1°C, respectively. Total annual precipitation is 906 mm, of which 314 mm falls as snow. There are 1,400 and 1,169 growing degree‐days above 5°C in the mixedwood and coniferous forests, respectively (Environment Canada, 2009).

A total of 7,010 sample plots (400 m^2^ each) with no major disturbance were provided by the *Ministère de la Forêt, de la Faune et des Parcs du Québec* (MFFPQ) and used to test the natural regeneration of balsam fir and black spruce in the mixedwood and coniferous bioclimatic domains (Figure 1, Methot et al., 2014). Overall, balsam fir predominantly occurred in the mixedwood domain, whereas black spruce showed the opposite pattern, with greater occurrence in the coniferous domain. For each sample plot, the number and DBH (diameter at breast height) of balsam fir and black spruce saplings (2 cm ≤ DBH <10 cm) and all mature trees of the sample plot, regardless of species (DBH ≥ 10 cm), were retrieved from the database, together with latitude, longitude, elevation, and soil type (clay or till, which are the dominant parent materials in both bioclimatic domains). We calculated the basal area (m^2^/ha) of mature trees in each plot and transformed this to a percentage of total mature trees. A threshold of 60% black spruce was used to classify sites that were developing toward black spruce dominance. This threshold was established according to the competitive ability and shade‐tolerance of the two species; balsam fir is generally more competitive and more shade‐tolerant than black spruce under similar growing conditions (Bakuzis & Hansen, 1965; Burns & Honkala, 1990). When the proportion of balsam fir was ≥40% of the coniferous component, the site was considered to be developing toward balsam fir dominance. We excluded from the analyses any disturbed sites, sites with <20% balsam fir or spruce in the canopy or regeneration layers, and jack pine sites (Messaoud, Bergeron, & Leduc, 2007).

### 2.2 Climate variables

Latitude, longitude, and elevation of each sample plot were used to derive climate attributes using the BIOSIM11 modelling software (https://cfs.nrcan.gc.ca/projects/133). Climate attributes included cumulative growing degree‐days >5°C (GDD_5) and total summer precipitation (May to August, PP_MA, mm). The GDD_5 threshold represents the base temperature above which plant growth begins. Averages of climate attributes corresponding to climate normals for the period 1981–2010 were used to determine the influence of climate on regeneration (Régnière, Saint‐Amant, & Béchard, 2017).

### 2.3 Statistical analyses

Statistical analyses were performed using the MIXED procedure of the SAS version 9.1 (Littell, Milliken, Stroup, & Wolfinger, 1996) testing the following model on balsam fir and black spruce, respectively:

(1) $$Y_{\mathrm{ijk}}=\beta_{0}+\beta_{1}F_{i}+\beta_{2}P_{\mathrm{ij}}+\beta_{3}S_{i}+\beta_{4}D_{\mathrm{ijk}}+\beta_{5}S\_{\mathrm{BA}}_{\mathrm{ijk}}+\beta_{6}T\_{\mathrm{BA}}_{\mathrm{ijk}}+\beta_{7}F_{i}D_{\mathrm{ijk}}S\_{\mathrm{BA}}_{\mathrm{ijk}}+\beta_{8}F_{i}D_{\mathrm{ijk}}{\mathrm{TBA}}_{\mathrm{ijk}}+\varepsilon_{\mathrm{ijk}},$$

where *Y* is the dependent variable representing the sapling number for the *i*th bioclimatic domain, *j*th sample plot and the *k*th species. The independent fixed effect variables are *F* for the bioclimatic domain, *P* for sample plot, *S* for species, *D* for soil type, and S_BA for parental tree basal area. To account for total basal area of a given sample plot, which may influence regeneration in some manner, we included total sample plot basal area (T_BA) as a covariate. To assess how regeneration was influenced by a combination of multiple site factors, the model also included interactions between bioclimatic domain, soil type and parental trees or total basal area. The model intercept is *β*_0_, while *β*_1_ to β_7_ are the parameters that were to be estimated for the independent fixed effects and their interactions. The error term, *ε_ijk_*, was assumed to be normally distributed (ε*_ijk_* ~ *N*(0, σ^2^)), with a mean of zero. Prior to analyses, and to meet the assumption of homoskedasticity of the residuals, sapling number and basal area were subjected to natural logarithmic transformation. The main categorical effects as bioclimatic domain, species and soil type were estimated using the PDIFF option of the LSMEANS statement. The interaction effects that included categorical and continuous variables were assessed by contrast analyses using the ESTIMATE statement. Overall, an effect was considered significant for *p* < 0.05 based on *t*‐tests of the fixed effects. To test the effect of climate on regeneration, relationships between regeneration and total summer precipitation and growing degree‐days >5°C were obtained using correlation analyses.

For a given species, an index that was termed “anomaly of sapling abundance” (*A*_SA_) was computed at the sample plot scale, as the difference between plot sapling abundance (SA_P_) and mean sapling abundance of the total study area (SA_M_). Thereafter, *A*_SA_ was plotted against the percentage basal area of parental trees (*S*_BA_) to derive a threshold of percent parental tree basal area from which a species maintains itself within the overall mean of the total study area (same value as the mean) or “overflows” (above this mean). A similar procedure was performed at the level of bioclimatic domain and another one controlling for both bioclimatic domains and soil type, using the following equation:

(2) $$\left\{\begin{array}{c} A_{\mathrm{SA}}=a+bS\_BA\\ {\mathrm{SA}}_{P}={\mathrm{SA}}_{M}\Rightarrow S_{\mathrm{BA}}=\mathrm{meantreshold}\\ {\mathrm{SA}}_{P}>{\mathrm{SA}}_{M}\Rightarrow S_{\mathrm{BA}}=\mathrm{overflow} \end{array}\right.$$

In Equation (2), the coefficients *a* and *b* are derived from a regression analysis.

## 3 RESULTS

Sapling abundance of balsam fir was significantly higher in the mixedwood than in the coniferous bioclimatic domain, while the converse was observed for black spruce (Table 1). Furthermore, this trend was also the same for both species regardless of soil type. Balsam fir sapling abundance was higher on till than on clay soils in mixedwood, while it was similar within the coniferous bioclimatic domain. Conversely, sapling abundance of black spruce was higher on clay than on till in both bioclimatic domains.

**Table 1:** Sapling abundance of balsam fir and black spruce (means, standard errors in parentheses) according to bioclimatic domain and soil type

Species	Soil type	Bioclimatic domain	
		Mixedwood	Coniferous
Balsam fir	Clay	1264.86^Aa^ (91.06)	625.52^Ba^ (80.44)
	Till	1682.75^Ab^ (54.75)	620.36^Ba^ (45.87)
	Total	1598.73^A^ (47.48)	621.68^B^ (39.86)
Black spruce	Clay	2511.30^Aa^ (119.82)	3925.72^Ba^ (189.76)
	Till	2079.37^Ab^ (49.50)	2864.29^Bb^ (75.49)
	Total	2166.21^A^ (46.38)	3137.25^B^ (74.83)

Superscripts indicate nonsignificant (same letter) or significant (different letters) differences between mixedwood and coniferous domains. Uppercase letters are comparisons between bioclimatic domains, while lowercase letters are comparisons between soil types within bioclimatic domain

### 3.1 Parental trees and species composition effects on regeneration

Overall, balsam fir and black spruce sapling abundance were significantly influenced by their parental trees and by total plot basal areas, while bioclimatic domain exhibited no significant effect (Table 2). Further, soil type did not influence balsam fir sapling abundance, whereas it significantly effected black spruce sapling abundance. The interaction between species basal area, bioclimatic domain and soil type was marginally significant for balsam fir, while the interaction between total basal area, bioclimatic domain and soil type were significant for black spruce.

**Table 2:** The table summarizes *F* tests of FIXED EFFECTS of the species saplings abundance (ln(stems/ha)) and the explanatory variables

Species	Effect	*F*‐value	*p*‐Value
Balsam fir	*R* ^2^ = 0.115		
	Bioclimatic domain	2.25	0.134
	Parental tree_BA	79.11	<0.001
	Total_BA	20.90	<0.001
	Soil type	0.01	0.938
	Species_BA × Bioclimatic domain × Soil type	2.25	0.055
	Total_BA × Bioclimatic domain × Soil type	1.14	0.330
Black spruce	*R* ^2^ = 0.128		
	Bioclimatic domain	0.78	0.337
	Parental tree_BA	146.94	<0.001
	Total_BA	221.19	<0.001
	Soil type	32.72	<0.001
	Species_BA × Bioclimatic domain × Soil type	1.58	0.192
	Total_BA × Bioclimatic domain × Soil type	4.14	0.006

BA: basal area.

**Figure 2:**
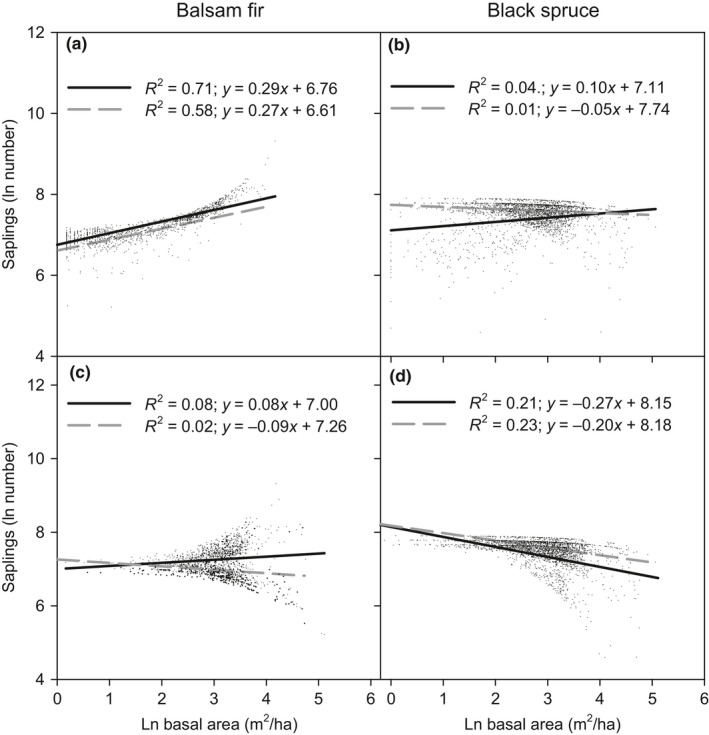
Abundance of balsam fir and black spruce saplings (ln(stems/ha)) with basal area for parental trees (a and b) and for the stand (c and d), according to bioclimatic domain. Black solid and gray dashed lines indicate the mixedwood and coniferous bioclimatic domains, respectively

In both bioclimatic domains, balsam fir sapling abundance not only increased with increasing parental tree basal area, but consistently maintained a higher sapling abundance in the mixedwood than in coniferous domain, although the model showed no significant difference (Table 1). Also, the rate of sapling abundance increased, given that the parental tree increase was stronger in the mixedwood compared to the coniferous domain, as shown by the significantly higher slope in the mixedwood bioclimatic domains (*p = *0.05; Figure 2a). Although no significant effect was observed in the coniferous domain, black spruce and balsam fir saplings decreased with decreasing the parental trees and total basal area, respectively (Figure 2b,c). For black spruce and regardless of bioclimatic domain, the relationship between sapling abundance and total basal area was significantly negative, but significantly less pronounced in the coniferous compared to the mixedwood domain (Figure 2d).

**Figure 3:**
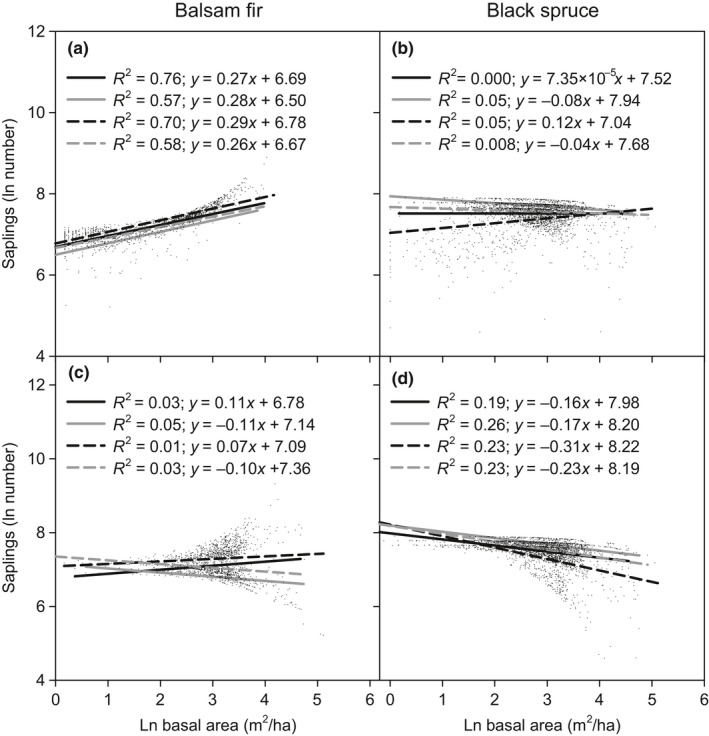
Abundance of balsam fir and black spruce saplings (ln(stems/ha)) with basal for parental trees (a and b) and for the stand (c and d), according to bioclimatic domain and soil type. The black solid and dashed lines respectively indicate clay and till soils in the mixedwood domain, while the gray solid and dashed lines respectively indicate clay and till soils in the coniferous domain

Balsam fir sapling abundance and parental tree basal area exhibited a positive relationship on both clay and till soils, regardless of bioclimatic domain (Figure 3a). We noted that in the mixedwood bioclimatic domain, sapling abundance was higher on till compared to clay soils, although the model indicated no significant relationship. In addition, on clay soils, the relationship between saplings abundance and parental tree basal area was stronger in the mixedwood in compared to the coniferous bioclimatic domain. For black spruce, a decrease in sapling abundance was associated with increasing parental tree basal area, except on till and marginally on clay soils in the mixedwood bioclimatic domain, where the relationship between sapling abundance and parental tree basal area was positive (Figure 3b). In both mixedwood and coniferous bioclimatic domains, significant higher regeneration for parental trees increased on clay soils (*p < *0.001). In the mixedwood bioclimatic domain, the relationship between sapling abundance and parental tree basal area was also stronger on clay soils. When both bioclimatic domains were contrasted, higher black spruce sapling abundance occurred in the coniferous domain (*p = *0.027). For balsam fir and irrespective of soil type, total basal area positively affected sapling abundance in the mixedwood, but negatively affected it in the coniferous bioclimatic domain; however, the overall difference remained nonsignificant (Figure 3c). For black spruce, the relationship between sapling abundance and total basal area was negative, regardless of bioclimatic domain or soil type (Figure 3d). The negative effect of total basal area on regeneration appeared to be significantly stronger on till soils in the mixedwood (*p* < 0.001) and coniferous (*p = *0.003) bioclimatic domains. In contrasting both bioclimatic domains, the negative effect of total basal area on black spruce sapling abundance was stronger in the coniferous than in the mixedwood bioclimatic domain, whether the soils were clay or till (*p < *0.001).

### 3.2 Anomaly of the sapling abundance

**Figure 4:**
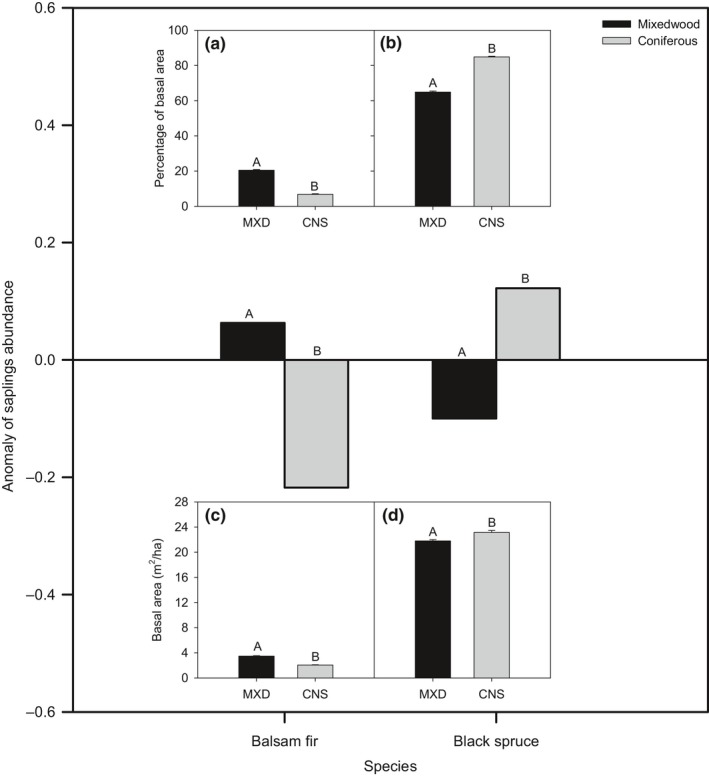
Anomaly of balsam fir and black spruce sapling abundance (ln(stems/ha)), according to bioclimatic domain. The anomaly represents the deviation from mean abundance of saplings for each species across the entire study area (positive = sapling excess, negative = sapling deficiency). Inset panels indicate percentage of basal area between the two bioclimatic domains (MXD = mixedwood, CNS = coniferous) for parental trees of balsam fir (a), black spruce (b), and the absolute values of the basal area for other tree species (c), and for the stand (d). The black and gray histograms indicate the mixedwood and coniferous domains, respectively. The letter above each histogram bar indicates nonsignificant (same letter) or significant (different letters) differences between mixedwood and coniferous bioclimatic domains

The anomaly of balsam fir regeneration abundance, representing the deviation from the mean of the total sapling abundance of the study area, was significantly positive in the mixedwood and negative in the coniferous domains, respectively (Figure 4). Conversely, the abundance of black spruce saplings was significantly positive in the coniferous and negative in the mixedwood bioclimatic domains.

At a bioclimatic domain scale, the basal area of parental trees was significantly higher in the mixedwood bioclimatic domain for balsam fir, in contrast with black spruce sites, which exhibited significantly higher parental tree basal area in the coniferous compared to the mixedwood bioclimatic domain (*p* = 0.050, Figure 4a,b). For both balsam fir and black spruce, the density of other deciduous and coniferous species, together with total stand densities, was higher in the mixedwood than in the coniferous bioclimatic domain ((Figure 4c–f).

**Figure 5:**
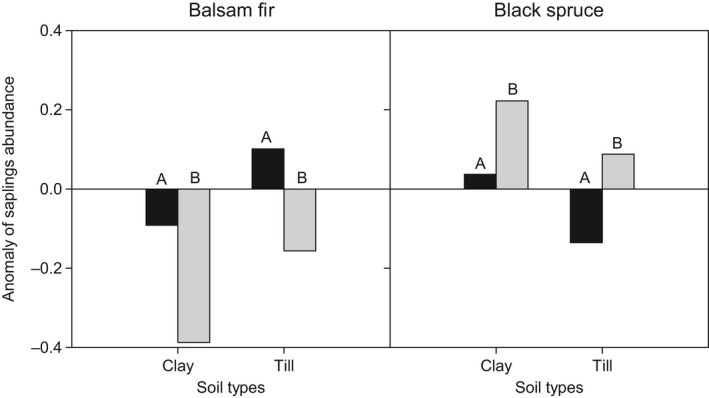
Anomaly of balsam fir and black spruce sapling abundances according to bioclimatic domain and soil type. The anomaly represents the deviation from the mean sapling abundance of each species within the whole study area (positive = sapling excess, negative = lack of saplings). The letter above each bar indicates nonsignificant (same letter) or significant (different letters) differences between mixedwood and the coniferous bioclimatic domains. Same legend as Figure 4

For balsam fir, when controlling for soil type, the anomaly of sapling abundance was negative on clay soils, and of a significantly greater magnitude (viz., a 4‐fold difference) in the coniferous bioclimatic domain (Figure 5). On till soils, the anomaly was positive in the mixedwood, but became negative and significantly stronger in the coniferous bioclimatic domain. For black spruce, the anomaly of sampling abundance was positive on clay soils in either domain, and of greater magnitude (a 5‐fold difference) in the coniferous than in the mixedwood bioclimatic domain (Figure 5). On till soils, black spruce regeneration was negative in the mixedwood and positive in the coniferous bioclimatic domain, and of greater magnitude in the mixedwood bioclimatic domain.

### 3.3 Regeneration at the population scale

**Figure 6:**
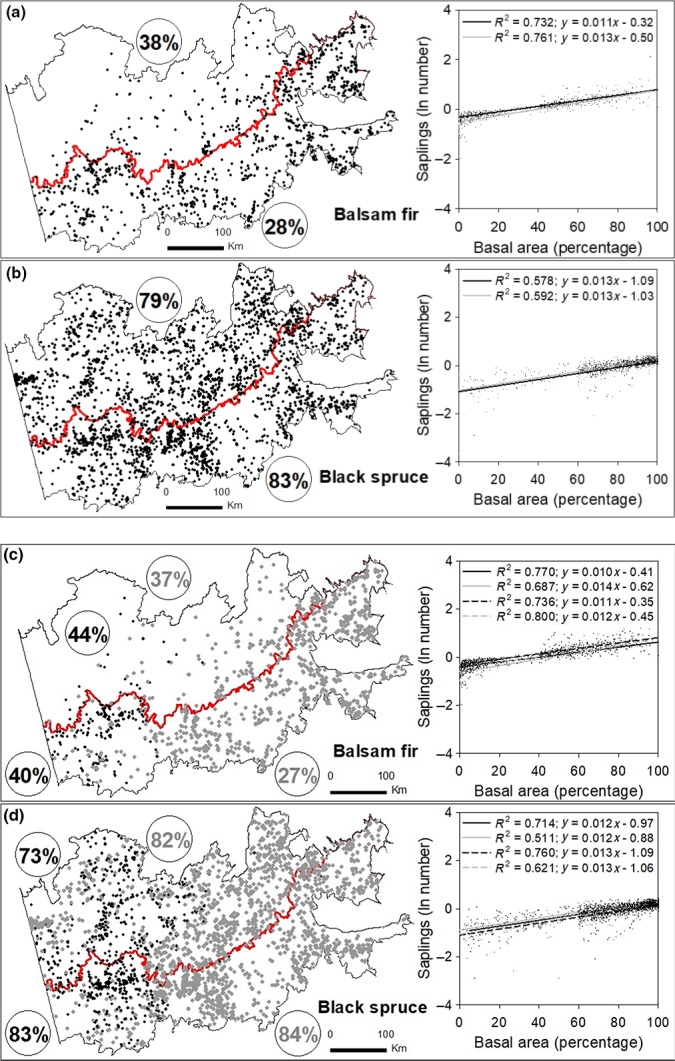
Maps of site distributions with percent of parental tree basal area that was required to provide a given species with a regeneration rate at least equal to mean regeneration for the entire study area (see Equation 2). Black and gray circled values pertain to forest domain (a, b), and the respective clay and till soils (c, d) inside a given forest domain. Scatter plots and trend lines depict relationships between the anomaly of sapling abundance and percent basal area of parental trees. Same legend as Figure 3

The relationship between the regeneration anomaly of balsam fir and black spruce and their respective percent parental tree basal areas allowed us to estimate a threshold of parental tree basal area that was required for a species to maintain its regeneration abundance within the mean regeneration of the entire study area (Figure 6). To maintain mean regeneration (1,319 saplings/ha), balsam fir required contributions from at least 28% of parental tree basal area in the mixedwood, and 38% in the coniferous bioclimatic domain. Sites meeting these requirements for balsam fir were predominantly found in the mixedwood bioclimatic domain. Black spruce maintenance of mean regeneration (1,772 saplings/ha) required at least 83% of parental tree basal area in the mixedwood, and 79% in the coniferous bioclimatic domain. Black spruce plots meeting this threshold apparently were similarly widespread across both bioclimatic domains. On clay soils, balsam fir required, to maintain mean regeneration, at least 40% and 44% of parental trees in the mixedwood and coniferous bioclimatic domains, respectively. However, on till soils, balsam fir only required 27% and 37% of parental trees in the mixedwood and in the coniferous domain, respectively. For black spruce, 82%–84% of parental trees were required to maintain mean regeneration, except on clay soils in the coniferous bioclimatic domain (73%).

The spatial pattern of species regeneration showed that balsam fir regeneration decreased with increasing distance (increasing latitude) from the ecocline (red line, Figure 1) in mixedwood bioclimatic domain, whereas this regeneration declined with increasing distance from the ecocline (higher latitude) in the coniferous bioclimatic domain (Table 3). In contrast, we observed no significant effect of distance for black spruce regeneration in the mixedwood bioclimatic domain, while regeneration did increase with distance in coniferous bioclimatic domain. Controlling for soil type, the effect of the distance from the ecocline did not affect balsam fir regeneration on clay, while it increased with increasing distance from the ecocline in the mixedwood bioclimatic domain on till soils. Yet, regeneration decreased with increasing distance in the coniferous bioclimatic domain, regardless of soil type. Black spruce regeneration was not affected by distance in the mixedwood domain, while it increased with increasing distance in the coniferous domain, regardless of soil type (Table 3).

**Table 3:** Product‐moment correlations (*r*) between species sapling abundance and latitude, representing the distance from the ecocline mixedwood/coniferous bioclimatic domains according to soil type

Species	Soil type	Latitude	
		Mixedwood	Coniferous
Balsam fir	Clay	−0.014	−0.166**
	Till	**0.048**	−0.123**
	Total	0.036**	−0.131**
Black spruce	Clay	−0.046	0.227**
	Till	−0.012	0.121**
	Total	−0.014	0.154**

Correlations that significantly different from zero (p < 0.05) are shown in boldface type: ^**^p < 0.001.

### 3.4 abundance and latitude, representing the distance fromClimate effects on regeneration

Cumulative growing degree‐days (GDD_5) exhibited a significant positive relationship with balsam fir regeneration on clay soils (Table 4). Although the relationship was also positive on till soils, the response was weaker than on clay soils. Total summer precipitation (PP_MA, May‐August) showed a positive significant relationship with balsam fir regeneration on till, while it was negative on clay soils. For black spruce, GDD_5 and PP_MA had negative significant effects on sapling abundance regardless of soil type, except for the nonsignificant effect of PP_MA on sapling abundance on clay soils (Table 4). The GDD_5 by PP_MA interaction showed respectively significant positive and negative effects upon balsam fir and black spruce regeneration. This relationship remained respectively positive and negative for balsam fir and black spruce regeneration, regardless of soil type.

**Table 4:** Product‐moment correlations (*r*) between species saplings abundance and climate variables according to the soil type

Species	Soil type	GDD_5	PP_MA	GDD_5 * PP_MA
Balsam fir	Clay	0.242**	−0.066*	0.175**
	Till	**0.034**	0.117**	0.093**
	Total	0.072**	0.095**	0.104**
Black spruce	Clay	−0.220**	0.023	−0.179**
	Till	−0.064**	−0.136**	−0.124**
	Total	−0.092**	−0.116**	−0.130**

Note

GDD_5, growing degree‐days >5°C; PP_MA, total summer precipitation between May and August (mm). We also include *r* for abundance versus the interaction between the two climate variables.

Correlations that significantly different from zero (*p* < 0.05) are shown in boldface type: *p < 0.01; **p < 0.001.

## 4 DISCUSSION

Our results demonstrated that parent trees played a significant positive role as seed sources on regeneration abundance of balsam fir and black spruce (Table 2; Figure 2a,b). Balsam fir parental tree basal area was higher in the mixedwood than in the coniferous bioclimatic domain, while the opposite was true for black spruce, the patterns of which overlapped with regeneration abundance (Figure 4). The presence of nearby seed trees has been previously reported as an important factor explaining regeneration abundance (Galipeau, Kneeshaw, & Bergeron, 1997; Redmond & Kelsey, 2018; Rossi, Morin, Gionest, & Laprise, 2012; Uprety, Asselin, Bergeron, & Mazerolle, 2014). Our study shows that for similar parental tree basal area between both bioclimatic domains, there was more regeneration for balsam fir in the mixedwood domain, while black spruce showing higher regeneration in the coniferous domain. In addition to parental tree effects on regeneration abundance, their seed production potential may also play a crucial role in regeneration. Balsam fir of comparable basal area has been shown to produce fewer seeds in the coniferous than in the mixedwood bioclimatic domain (Messaoud, Bergeron, & Asselin, 2007), resulting in lower subsequent regeneration in the coniferous domain. In the same study, Messaoud, Bergeron, and Asselin (2007) found that black spruce showed similar seed production between the two bioclimatic domains. Therefore, higher regeneration in the coniferous domain might only be linked with site species composition, local climate, or soil characteristics that facilitate higher regeneration. The tight link between parental trees and regeneration for balsam fir in one hand, and the observed discrepancy that was noted for black spruce on the other, suggests that balsam fir is more dependent on parental tree proximity than is black spruce. Unlike large balsam fir seeds that fall beneath or close to their parent tree, the small size of black spruce seeds allows them to disperse for greater distances (effective distances of 20–80 m; Asselin, Fortin, & Bergeron, 2001; Bakuzis & Hansen, 1965; Fryer, 2014) from the parent tree. The basal area of other species on sites that are predominantly composed of balsam fir or black spruce can serve as a proxy for forest composition, which may influence sapling abundance to some extent. Indeed, basal area of other tree species, which when these are dominated by deciduous species, was higher in the mixedwood than in the coniferous bioclimatic domain (Figure 2c, Appendices S1 and S2). The presence of deciduous species, such as paper birch or trembling aspen, which are two dominant deciduous species in our study area, may promote suitable conditions (e.g., higher soil temperatures, increased organic layer decomposition, and impeded paludification), thereby increasing sapling survival, especially for balsam fir (Arbour & Bergeron, 2011; Kabzems, Comeau, Filipescu, Rogers, & Linnell Nemec, 2016). Also, large balsam fir seeds can be protected from seed predation and competition with herbaceous plants by a broadleaf litter layer (Bürzle et al., 2018; Facelli & Pickett, 1991; Garcia et al., 2002; Kellner & Swihart, 2016). In contrast, thicker broadleaf litter layers appear to be detrimental for small seeded tree species, such as black spruce, because their seeds contain fewer nutritional reserves for germination and sufficient root elongation to penetrate the mineral soil through the litter layer (Asplund, Hustoft, Nybakken, Ohlson, & Lie, 2018; Wang & Kemball, 2005). Thus, the lower density of other species in the coniferous domain could trigger a population shift from warmer balsam fir conditions to colder conditions to which black spruce and associated species are better adapted (Bakuzis & Hansen, 1965). We would posit that a higher diversity of forest composition exerts two major effects upon the regeneration: facilitation for balsam fir, and exclusion for black spruce. Yet, the effects of exclusion were linked more indirectly to the negative effects of broadleaf litterfall on black spruce regeneration, as previously mentioned.

The total basal area of all species in a given plot did not significantly influence balsam fir sapling abundance, while it showed a negative effect on black spruce regeneration (Figure 2c,d). An increase in stand density decreases the amount of light reaching the understory where saplings are mostly found (cf. gaps or cutovers). Since balsam fir is more shade‐tolerant than is black spruce, the increase in stand density and subsequent decrease in understory light would explain negative effects that exerted on black spruce and the mild influence on balsam fir sapling abundance (Asplund et al., 2018; Burns & Honkala, 1990). In addition, black spruce sapling abundances declined more strongly in the mixedwood than in the coniferous bioclimatic domain, which suggests a possible increase in competition with coexisting shade‐tolerant species.

Soil type effects on regeneration resulted in higher balsam fir sapling abundances on till, contrasting with better black spruce regeneration that occurred mostly on clay. For balsam fir, the negative effects of clay soils on sapling abundance were stronger in the coniferous bioclimatic domain, while positive effects of clays soils only occurred in the mixedwood bioclimatic domain. For black spruce, positive effects of clay soils on sapling abundance showed a stronger response in the coniferous bioclimatic domain, while the effects of till soils were negative in the mixedwood and positive in the coniferous bioclimatic domain. The negative effect of clay soils on balsam fir has already been reported in a previous research (Messaoud, Bergeron, & Leduc, 2007). Negative effects of clay soils on balsam fir may be due to higher water content that often characterize clay soils, which have lower temperatures and lower rates of evaporation. Balsam fir saplings on clay are more likely to experience oxygen‐deprivation in the rooting zone due to waterlogging and reduced gas exchange, conditions that are predominantly found in the coniferous domain. It has been further reported that lower temperatures in clay soils are likely to favor increased organic matter accumulation, which is unfavorable for balsam fir establishment and survival (Fenton, Lecomte, Legare, & Bergeron, 2005; Messaoud, Bergeron, & Leduc, 2007; Terrier et al., 2015).

When parental tree basal area is lower, higher black spruce regeneration occurs in the coniferous domain on clay soils and is lower on till soils in the mixedwood domain. For increasing basal area of parental trees, differences in black spruce regeneration between forest domains and soil type decreased to negligible levels at higher parental tree basal areas (Figure 3b). This suggests that the effect of soil type on black spruce regeneration greatly diminishes as parental tree basal area increases, likely due to convergence of soil temperature and organic layer thickness conditions between forest domains and soil type. Thus, dominant black spruce stands may modify their own microclimate and soil conditions by increasing soil moisture and lowering soil temperature on till soils, which is favorable for promoting their sapling abundance. However, on clay soils occurring in the coniferous bioclimatic domain, black spruce regeneration is more likely to be negatively affected, due to soil moisture saturation and soil temperature decrease, which both lead to paludification (Fenton et al., 2005; Terrier et al., 2015).

To maintain its regeneration abundance within the mean of the whole study area (1,319 saplings/ha), balsam fir required at least 28% of parental tree basal area in the mixedwood, and 38% above their northern limit, that is, in the coniferous bioclimatic domain. However, black spruce required at least 79% and 83% of parental tree basal area to maintain regeneration within the mean of the study area (1,772 saplings/ha) within the coniferous and mixedwood domains, respectively. In the coniferous domain, balsam fir regeneration requires more parental trees to compensate for lower balsam fir seed production, which in turn is likely to lower regeneration abundance. In accounting for soil type, balsam fir must maintain regeneration within the mean of the total study area, given that there are fewer parental trees on till than on clay, regardless of the forest domain. This contrasts with black spruce, which requires higher numbers of parental trees than does balsam fir. This can be explained by lower black spruce seed inputs compared to those of balsam fir, due the smaller semi‐serotinous cones that are form an aerial seed bank for former, which can release small quantities of seed continuously but episodically with the occurrence of fire (Messaoud, Bergeron, & Asselin, 2007; Rossi, Morin, Gionest, & Laprise, 2017). Another explanation may be related to higher seedling mortality within the low light understory for the less shade‐tolerant black spruce. This may explain why black spruce must maintain regeneration abundance within the mean of the study site, where parental tree basal areas are similar irrespective of forest domains and soil type.

The spatial pattern of balsam fir regeneration revealed better performance closer to the ecocline (red line, Figure 1) in the mixedwood, whereas it declined further from this limit in coniferous bioclimatic domain (Table 3). The progressively lower performance of balsam fir regeneration throughout the south and further north of the ecocline was related to the decline in the presence of parental trees (*p < *0.0001). Black spruce regeneration did not display any trend in the mixedwood, but it increased further north in coniferous bioclimatic domain. This finding was also related to the absence spatial patterning of parental tree abundance (*p* > 0.05) in the mixedwood, while the abundance of parental trees increased further north of the ecocline (*p = *0.004). Controlling for soil type, the general spatial pattern of species regeneration was also related to the abundance of parental trees. Yet, we found that regeneration was not affected by distance from the ecocline for balsam fir and black spruce on clay in the mixedwood bioclimatic domain. Surprisingly, this response was not related to parental trees, since they increased approaching the ecocline (*p = *0.007 and *p* < 0.0001 for balsam fir and black spruce, respectively). Further, black spruce regeneration increased farther north of the ecocline, although basal area of parental trees remained constant, regardless of soil type (*p* > 0.05, Table 3). These unexpected results could be related to other factors, such as competition between saplings, which could play a big role in counterbalancing the influence of parental trees. Indeed, Messaoud, Goudiaby, and Bergeron (2019) found higher mortality of balsam fir seedlings (first stage of regeneration) on clay in the mixedwood domain compared to the coniferous domain and pointed out the role of competition between seedlings. We had previously mentioned the negative effect of deciduous tree species on black spruce regeneration. Therefore, the respective spatial increase and decrease in deciduous tree species closer to (*p = *0.0006) and further north (*p < *0.0001) of the ecocline may play an additional role in black spruce regeneration.

The effect of the climate on balsam fir regeneration showed a stronger relationship with GDD_5 than did black spruce (Table 4), illustrating greater adaptation to warmer environments that was shown by balsam fir compared to black spruce (Bakuzis & Hansen, 1965). Furthermore, balsam fir regeneration had a stronger positive relationship with GDD_5 on clay than on till soils. Clay soils are known to be colder, with a greater water‐holding capacity than tills (Fenton et al., 2005; Terrier et al., 2015), while till soils are not only warmer, but are subject to greater rates of moisture evaporation (Kersten, 1952). This explains why a negative relationship was found between balsam fir sapling abundance and summer precipitation (PP_MA) on clay soils, while a positive relationship was demonstrated for till soils. For black spruce, GDD_5 had a negative effect on seedling abundance on both till and clay, most like due to lower tolerance to higher temperatures than balsam fir, which are found mostly in southern locations. Also, the effect of PP_MA on black spruce regeneration was not significant on clay, but significantly negative on till soils. On clay soils, black spruce demonstrated its adaptation to cold soil temperatures and higher water content (Trugman et al., 2016). On till soils, the negative relationship between PP_MA and black spruce regeneration may be due to competition for water with coexisting deciduous species, since till soils are subject to greater water drainage and higher rates of evaporation. Further, the interaction between temperature and precipitation can be used to indicate the effects of drought (Sigdel et al., 2018). Thus, we used the interaction between GDD_5 and PP_MA as its proxy. The interaction was significantly positive and negative for balsam fir and black spruce regeneration, respectively. This effect remained the same regardless of soil type (Table 4), indicating that drought did not negatively affect balsam fir regeneration, while “drought” certainly did affect black spruce. Thus, balsam fir appears to be more drought tolerant than black spruce, which could be related to adaptation of balsam fir to warmer conditions compared to cooler and moister conditions for black spruce (Bakuzis & Hansen, 1965). Furthermore, the positive effect of drought on balsam fir regeneration was more obvious on clay than on till soils (*r* = 0.175 vs. 0.093), indicating that balsam fir was less adapted to the occurrence greater soil moisture levels. Another explanation is that since balsam fir is more shade‐tolerant than black spruce (Bakuzis & Hansen, 1965), black spruce regeneration occurs under lower forest cover and, thus, is more exposed to drought conditions (Harmon, 1987; Kellner & Swihart, 2016; Oleskog & Sahlen, 2000; Redmond & Kelsey, 2018). Levels of PP_MA were equivalent across the study area, but drought appeared to occur more frequently in the warmer mixedwood than in the cooler coniferous bioclimatic domain (Appendix S3).

The ecocline that exists between the mixedwood and coniferous bioclimatic domains of eastern North America represents a shift from balsam fir to black spruce dominance. This ecocline is not the northern limit of balsam fir, which extends further north (54°; Sirois, 1997). This explains the occurrence of scattered balsam fir populations in the coniferous bioclimatic domain, where lower regeneration did not compromise the stability of such populations, just as long as a minimum parental tree basal area remained to maintain mean regeneration. The insight provided by the current study agrees with previous results showing that the few balsam fir populations found in the coniferous domain apparently can persist for a long time in the absence of severe disturbance (Sirois, 1997).

Catastrophic wildfire is a major disturbance in the boreal forest, which can exert a strong influence on vegetation composition and dynamics at any given location (Bergeron, Gauthier, Flannigan, & Kafka, 2004; Lefort, Leduc, Gauthier, & Bergeron, 2004; Portier, Gauthier, Leduc, Arseneault, & Bergeron, 2016). Balsam fir is a fire‐intolerant species because its thin bark offers weak protection against fires. Its low abundance has been reported as being related to large and intense fire regimes, since it cannot maintain a seedbank in the tree crown, unlike black spruce (Bakuzis & Hansen, 1965). Moreover, large fires would kill the parental trees of balsam fir, thereby negatively affecting its regeneration (Morgan, Vincent, & Camac, 2018). In contrast, black spruce is adapted to fire thanks to its thick bark, which offers efficient protection against fire. This adaptation partly explains why balsam fir and black spruce parental trees respectively decreased and increased further north of the ecocline (Table 3). Also, black spruce benefits more from large fires, owing to its aerial seed bank that persists in serotinous cones remaining in the canopy for many years, until a fire opens them to liberate seeds. If climate change progresses toward warmer conditions with no evidence of dryness (Bose, Weiskittel, & Wagner, 2017), large fires would be expected to be less frequent, to be replaced with small fires. Also, higher predicted temperatures are expected to stimulate seed supplies (van der Meer, Jorritsma, & Kramer, 2002; Messaoud, Bergeron, & Asselin, 2007; Roland, Schmidt, & Johnstone, 2014). In such a scenario, small fires would open black spruce forests by burning the organic matter layer, thereby promoting invasion by more warm‐adapted and shade‐tolerant balsam fir. Fir expansion would occur through higher seed inputs in the vicinity of balsam fir stands in the coniferous domain. To be effective, balsam fir invasion following small fires would be preceded by its establishment on suitable sites (Bose et al., 2017). Therefore, the dependence of regeneration on parental trees and site condition, as has been illustrated by the current research, would help to better address real effects of climate change and disturbance on vegetation composition and dynamics in the boreal forest.

## 5 CONCLUSIONS

The northern limit of balsam fir dominance (*ca* 49°N) and, therefore, the transition between the mixedwood and coniferous domains, can be linked to lower balsam fir regeneration in the coniferous compared to the mixedwood bioclimatic domain. Our results further showed that black spruce regeneration was less adapted to warmer and drier climate conditions than more shade‐tolerant balsam fir, explaining the dominance of the latter species in the mixedwood bioclimatic domain. To maintain regeneration equivalent to the mean for the entire study area, balsam fir required 10% more parental trees in the coniferous than in the mixedwood bioclimatic domain. This is more likely due to lower temperatures and shorter growing seasons in northern compared to southern sites. Summer precipitation appeared to be negligible, which may explain why balsam fir fails to occupy all potentially suitable sites in the coniferous bioclimatic domain. Furthermore, balsam fir regeneration was lower on clay than on till soils, indicating that lower soil temperatures coupled with higher water content occurring in clay soils are not suitable for balsam fir regeneration, especially in the coniferous bioclimatic domain. Nevertheless, the threshold of parental tree basal area that must be met for species regeneration to be similar to the mean study area is lower for balsam fir compared to black spruce, which explains why mixedwood balsam fir populations were found far above the ecocline between the two bioclimatic domains. The current research provides insight into the role of parental trees and site conditions on balsam fir and black spruce regeneration, and their influence on population dynamics within and outside of their respective mixedwood and coniferous bioclimatic domains. Balsam fir naturally occurs mostly in the southern mixedwood bioclimatic domain but reaches its northern limit in terms of dominance as has been generally depicted by the ecocline between mixedwood and coniferous bioclimatic domains. Yet, our research shows that this ecocline is not the limit of balsam fir in terms of species presence, as shown by patchy but viable balsam fir populations found inside the coniferous forest domain, even extending further northward. Another new insight provided by this study is the local and landscape spatial scale that was addressed, which considerably increases our understanding of vegetation dynamics in the boreal biome within the context of future global change.

## CONFLICT OF INTEREST

None declared.

## AUTHOR CONTRIBUTIONS

YM conceived the ideas and designed the experiments. VG conceived the analytical methodology and analysed the data. YM and VG wrote the manuscript and YB revised the previous drafts and made substantial comments.

## Data Availability

Data are available from the *Ministère de la Forêt de la Faune et des Parcs du Québec* (MFFPQ): https://geoegl.msp.gouv.qc.ca/igo/mffpecofor/.
