# Supplementary material for: Persistence of balsam fir and black spruce populations in the mixedwood and coniferous bioclimatic domain of eastern North America
Source: Ecol Evol. 2019 Apr 13;9(9):5118–32. doi: 10.1002/ece3.5069 (PMC6509386; doi:10.1002/ece3.5069)
Supplement: Supplementary file 1 [file ECE3-9-5118-s001.doc]

**SUPPORTING INFORMATION**

**Appendix S1. Composition of canopy species other than black spruce and balsam fir in the study area and their mean basal area (m^2^ ha^-^1) in each bioclimatic domain. Standard errors are included in parentheses.**

| **Species** | **Common name** | **Scientific name** | **Balsam fir stand** | | **Black spruce stand** | |
| --- | --- | --- | --- | --- | --- | --- |
|  |  |  | **Mixedwood** | **Coniferous** | **Mixedwood** | **Coniferous** |
| Deciduous | American mountain-ash | *Sorbus americana* Marsh | 0.051 (0.010) | 0.006 (0.003) | 0.000 (0.000) | 0.000 (0.000) |
|  | Balsam poplar | *Populus balsamifera* L. | 0.029 (0.016) | 0.000 (0.000) | 0.000 (0.000) | 0.000 (0.000) |
|  | Black cherry | *Prunus serotina* Ehrh. | 0.004 (0.004) | 0.000 (0.000) | 0.000 (0.000) | 0.000 (0.000) |
|  | Green alder | *Alnus viridis* (Vill.) Lam. & DC. | 0.021 (0.007) | 0.024 (0.008) | 0.000 (0.000) | 0.000 (0.000) |
|  | Grey alder^2^ | *Alnus incana* (L.) Moench | 0.213 (0.042) | 0.023 (0.013) | 0.000 (0.000) | 0.000 (0.000) |
|  | Grey birch | *Betula populifolia* Marsh. | 0.000 (0.000) | 0.003 (0.003) | 0.000 (0.000) | 0.000 (0.000) |
|  | Mountain maple | *Acer spicatum* Lam. | 0.020 (0.007) | 0.002 (0.002) | 0.000 (0.000) | 0.000 (0.000) |
|  | Northern mountain-ash | *Sorbus decora* (Sarg.) Schneid. | 0.000 (0.000) | 0.006 (0.003) | 0.000 (0.000) | 0.000 (0.000) |
|  | Paper birch^1 2^ | *Betula papyrifera* Marsh. | 3.932 (0.277) | 2.137 (0.200) | 1.178 (0.095) | 0.604 (0.065) |
|  | Pin cherry | *Prunus pensylvanica* L. f. | 0.089 (0.018) | 0.004 (0.004) | 0.000 (0.000) | 0.000 (0.000) |
|  | Red maple | *Acer rubrum* L. | 0.078 (0.024) | 0.000 (0.000) | 0.000 (0.000) | 0.000 (0.000) |
|  | Eastern shadbush | *Amelanchier canadensis* (L.) Medik. | 0.006 (0.003) | 0.002 (0.001) | 0.000 (0.000) | 0.000 (0.000) |
|  | Trembling aspen^1 2^ | *Populus tremuloides* Michx. | 1.025 (0.158) | 0.573 (0.119) | 0.505 (0.061) | 0.294 (0.043) |
|  | Willow^1 2^ | *Salix sp.* | 0.166 (0.028) | 0.051 (0.013) | 0.085 (0.012) | 0.052 (0.007) |
|  | Yellow birch | *Beyula alleghaniensis* Britt. | 0.092 (0.045) | 0.000 (0.000) | 0.000 (0.000) | 0.000 (0.000) |
| Coniferous | Jack pine | *Pinus banksiana* Lamb. | 0.398 (0.067) | 0.652 (0.171) | 1.026 (0.084) | 1.386 (0.094) |
|  | Northern white-cedar | *Thuja occidentalis* L. | 0.000 (0.000) | 0.010 (0.010) | 0.000 (0.000) | 0.000 (0.000) |
|  | Red spruce | *Picea rubens* *Sarg.* | 0.033 (0.020) | 0.026 (0.026) | 0.000 (0.000) | 0.000 (0.000) |
|  | Tamarack | *Larix laricina* (Du Roi) K. Koch | 0.149 (0.037) | 0.223 (0.087) | 0.278 (0.040) | 0.189 (0.034) |
|  | White pine | *Pinus strobus* L. | 0.004 (0.004) | 0.000 (0.000) | 0.005 (0.005) | 0.000 (0.000) |
|  | White spruce | *Picea glauca* (Moench) Voss | 1.136 (0.145) | 0.696 (0.144) | 0.232 (0.039) | 0.081 (0.022) |

**Appendix S2.** **Average proportion of deciduous species on sites that were dominated by balsam fir or black spruce regeneration in the mixedwood and coniferous bioclimatic domains. The uppercase superscript on each mean value indicates a non-significant (same letter) or significant (different letters) difference between mixedwood and coniferous bioclimatic domains. Standard errors are in parentheses.**

| Deciduous tree cover |  | Bioclimatic domain |  |
| --- | --- | --- | --- |
| Regeneration | Soil type | Mixedwood | Coniferous |
| Balsam fir | Clay | 74.81^A^ (3.02) | 61.63^B^ (5.69) |
|  | Till | 73.14^A^ (1.48) | 73.07^A^ (2.74) |
|  | *Total* | *73.56*^A^ (1.43) | *70.48*^A^ (*2.49*) |
| Black spruce | Clay | 55.11^A^ (3.24) | 42.37^B^ (3.38) |
|  | Till | 55.99^A^ (1.97) | 44.26^B^ (2.06) |
|  | *Total* | *55.75*^A^ (*1.68*) | *43.78*^B^ (*1.76*) |

**Appendix S3.** **Distribution of the climate variables in the study area: a) growing degree-days; and b) total summer precipitation (May-August).**

**
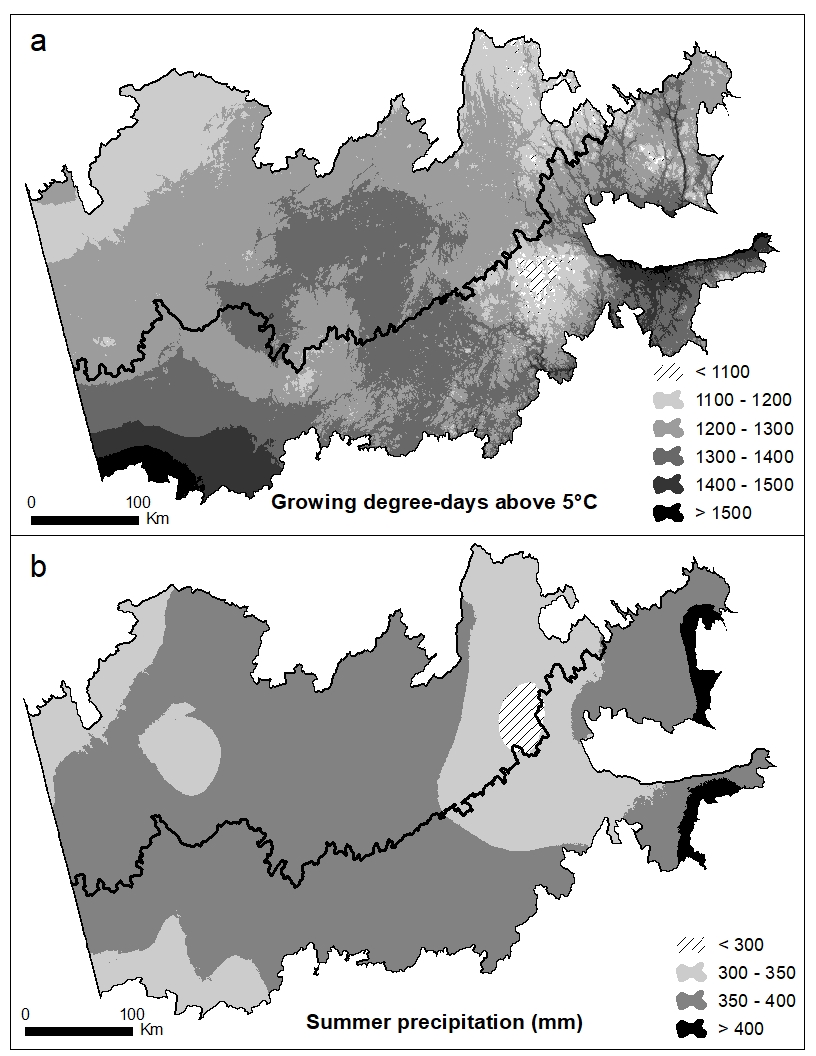
**
